# 46,XY disorder of sexual development resulting from a novel monoallelic mutation (p.Ser31Phe) in the steroid 5*α*-reductase type-2 (*SRD5A2*) gene

**DOI:** 10.1002/mgg3.76

**Published:** 2014-03-16

**Authors:** Bertha Chávez, Luis Ramos, Rita Gómez, Felipe Vilchis

**Affiliations:** 1 Department of Reproductive Biology, Instituto Nacional de Ciencias Médicas y Nutrición S. Z.México City, México; 2 Department of Clinical Epidemiology Medical Research Unit, Hospital de Especialidades, CMN Siglo XXI, Instituto Mexicano del Seguro SocialMéxico City, México

**Keywords:** 46,XY DSD, DHT, genital ambiguity, hypospadias, micropenis, SRD5A2

## Abstract

Inactivating mutations of the 5*α*-steroid reductase type-2 (*SRD5A2*) gene result in a broad spectrum of masculinization defects, ranging from a male phenotype with hypospadias to a female phenotype with Wolffian structures. Molecular studies of the *SRD5A2* revealed a new heterozygous gene variant within the coding region that results in phenotypic expression. A c.92C>T transition changing serine to phenylalanine at codon 31 of exon 1 (p.Ser31Phe) was identified in a patient with 46,XY disorder of sexual development who displayed glandular hypospadias with micropenis and bilateral cryptorchidism. The restoration of the p.Ser31Phe mutation by site-directed mutagenesis and transient expression assays using cultured HEK-293 cells showed that this novel substitution does not abolish but does deregulate the catalytic efficiency of the enzyme. Thus, the maximum velocity (*V*_max_) value was higher for the mutant enzyme (22.5 ± 6.9 nmol DHT mg protein^−1^ h^−1^) than for the wild-type enzyme (9.8 ± 2.0 nmol DHT mg protein^−1^ h^−1^). Increased in vitro activity of the p.Ser31Phe mutant suggested an activating effect. This case provides evidence that heterozygous missense mutations in *SRD5A2* may induce the abnormal development of male external genitalia.

## Introduction

The human 5*α*-steroid reductase type-2 (EC 1.3.99.5) encoded by the *SRD5A2* gene has a tissue-specific expression pattern. It is found predominantly in the stromal cells of internal and external reproductive organs. During embryogenesis, this isozyme plays a central role in the differentiation of the male phenotype by catalyzing the conversion of testosterone (T) to 5*α*-dihydrotestosterone (DHT), an androgen that is 10–15 times more potent than testosterone. DHT acts to drive the formation of the male urethra and prostate at the urogenital sinus and induces swelling and folding at the genital tubercle to form the penis and scrotum (Wilson et al. 1993). Acquired abnormalities in the 5*α*-reductase type-2 isozyme due to gene mutations are known to impair the catalytic efficiency of the enzyme and diminish the levels of DHT, which in turn results in a wide spectrum of clinical phenotypes (Sinnecker et al. 1996; Canto et al. 1997). Molecular genetics studies have demonstrated that inactivating mutations in *SRD5A2* leads to steroid 5*α*-reductase-2 deficiency, an autosomal recessive form of 46,XY disorder of sexual development (Wilson et al.^1993^; Russell et al. 1994). Males affected by this disorder usually present with ambiguous external genitalia, microphallus, perineoscrotal hypospadias, prostatic hypoplasia and cryptorchid or inguinal testes. Virilization and deepening of the voice occur at puberty along with penile enlargement and muscle-mass development without gynecomastia. These patients exhibit scarce facial and body hair and an absence of temporal male baldness, acne, and prostate enlargement, as these phenomena are dependent on the action of DHT (Mendonca et al. 2010). With the exception of a single case of uniparental disomy (Chávez et al. 2000), most patients with SRD5A2 deficiency reported thus far are homozygous (65%) or compound-heterozygous (35%) for loss-of-function mutations (Vilchis et al. 2010; Maimoun et al. 2011). However, in some described cases, only one affected allele is identified, suggesting that certain mutations may be dominant (Thigpen et al. 1992; Vilchis et al. 2010; Shabir et al. 2012). Here, we report a case of undervirilization associated with a monoallelic missense mutation in *SRD5A2* that causes an atypical enzymatic activity.

## Clinical Report

The present case describes an 18-year-old adolescent of Mexican–Mestizo origin with incompletely developed external genitalia who was initially referred to the Urology Department because of hypospadias. He had an endocrine assessment at the age of 8 years. There is no known history of consanguinity in his family. The patient was reared as a boy and now displays male sex identity. Physical examination at the time of endocrine assessment revealed the presence of bilateral inguinal testes (right gonad, 2.2 × 3 cm; left gonad, 2.0 × 3 cm) and a phallus of 2.0 cm in length presenting glandular hypospadias. A pelvic ultrasound showed no Müllerian structures. The karyotype was 46,XY. By the age of 9 years 5 months, the penis had grown to 2.5 cm and the testicles had descended into the scrotal sac. When testicular reserve was assessed using the hCG stimulation test, testosterone concentration had changed from 0.45 to 2.0 ng/mL. Treatment was recommended for phallic growth with a positive response. His sex hormone profile was as follows: FSH 2.5 IU/L (normal: 1.0–8.0 IU/L), LH 2.4 IU/L (normal: 0.5–7.0 IU/L), basal testosterone levels 19.2 nmol/L (normal: 12.5–70.0 nmol/L), and DHT 0.48 nmol/L (normal: 1.0–2.7 nmol/L). His levels of androstenedione and estradiol were 2.0 nmol/L (normal: 2.4–6.9 nmol/L) and 17.5 pg/mL (normal: <50 pg/mL), respectively. At the age of 18 years, the patient's penis measured almost 6 cm in length, and the testes were 4.5 × 2.5 cm, with Tanner stages completed. He did not present breast enlargement or facial fuzz. After the signing of informed consent forms, blood samples were obtained from the patient and his mother for genomic DNA extraction; the father was not available to participate in the molecular studies. The study protocol was approved by the Institutional Ethical Committee for Investigation in Humans (INCMNSZ).

## Methods

Coding sequence abnormalities in *SRD5A2* (NG_008365.1, [OMIM: 607306]) were assessed by exon-specific PCR, Single-Strand Conformation Polymorphism, and sequence analysis, using previously described specific primers and conditions (Vilchis et al. 2000). For functional assays, the commercial vector pCMV6-XL4 (OriGene Technologies Inc., Rockville, MD), containing the full-length human *SRD5A2* cDNA (clone #SC119922) was used as a template to synthesize constructs. The mutant vectors were created as previously described (Vilchis et al. 2008, 2010), using the Gene Tailor Site-Directed Mutagenesis System (Invitrogen, Life Technologies, Carlsbad, CA) and the following primers: 31F, 5′-CCTTGTACGTCG CGAAGCCCTTCGGCTACG-3′ (forward) and 31R, 5′-GGGCTTCGCGACGTACAAGGCCAG TGCCCC-3′ (reverse). Mutant and control DNAs were sequenced using an ABI-PRISM 3100 automated sequencer (Applied Biosystems, Foster City, CA). Determination of 5*α*-reductase activity in transfected cells was carried out as previously described (Vilchis et al. 2008). Molecular screening of the genes *NR3C4* (AR, NM_000044.2, [OMIM: 313700]), *HSD17B3* (NG_008157.1; [OMIM: 605573]), and *NR5A1* (SF1, NG_008176.1; [OMIM: 184757]) was performed using primers designed based on database sequences.

## Results

**Figure 1:**
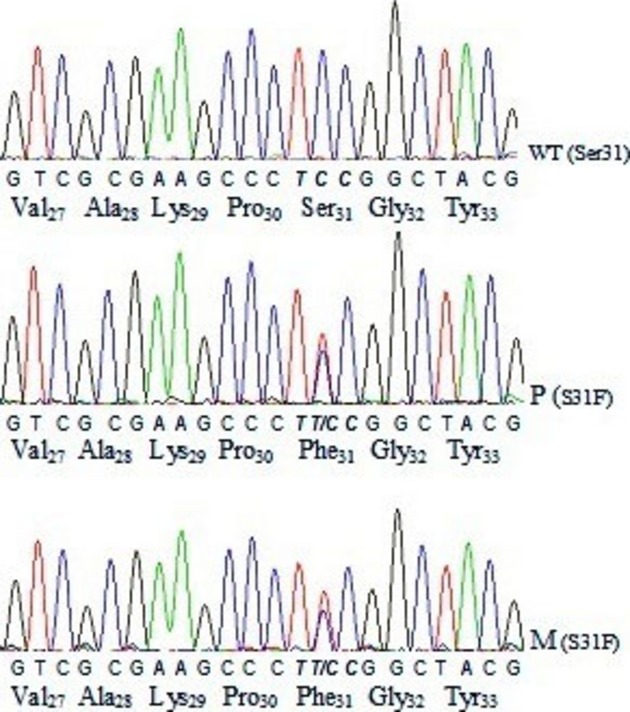
Partial nucleotide sequence of the *SRD5A2* gene showing a C→T heterozygous mutation (p.Ser31Phe) at exon 1 from a patient (P) with 46,XY DSD and his mother (M). Genomic DNA from a normal healthy male (WT) served as the control.

**Figure 2:**
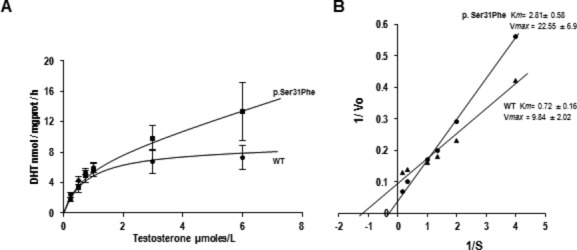
Characterization of 5*α*-reductase activity in cultured HEK-293 cells after transient transfection with normal (XL4) and mutant (S31F) *SRD5A2* cDNAs. Kinetic constants were assessed by in vitro enzymatic assays using whole cell sonicates and [^3^H]testosterone as a substrate. Reduction of different concentrations of testosterone to 5*α*-DHT in the presence of 0.5 mmol/L Nicotinamide Adenine Dinucleotide Phosphate (NADPH) (A). Lineweaver–Burk plots of activities of normal and mutant SRD5A2 proteins (B). The error bars represent the mean ± standard deviation from four independent reactions.

Single-Strand Conformation Polymorphism and sequencing analyses of the complete coding region, including the exon–intron boundaries of *SRD5A2*, revealed variations in exon 1 but not in exons 2–5. As shown in Figure 1, the DNA sequence of the mutant fragment showed a single-base mutation in exon 1. A c.92C>T transition was identified in codon 31. This missense mutation was responsible for a Ser→Phe substitution (codon TTC instead of TCC). Both the patient and his mother were heterozygous carriers for the same (p.Ser31Phe) mutation. To examine the biochemical consequences of this gene variant, it was recreated in SRD5A2 cDNA. As shown in Figure 2, the substitution of phenylalanine for serine at position 31 produces an enzyme with altered biochemical characteristics. For example, the apparent Michaelis–Menten constant (*K*_m_) for testosterone was higher for the mutant enzyme (2.8 ± 0.5 *μ*mol/L) than for the wild-type enzyme (0.7 ± 0.1 *μ*mol/L). Likewise, the maximum velocity (*V*_max_) value was higher for the mutant enzyme (22.5 ± 6.9 nmol DHT mg protein^−1^ h^−1^) than for the wild-type enzyme (9.8 ± 2.0 nmol DHT mg protein^−1^ h^−1^). Based on in vitro metabolic assay data, this mutation appears to increase enzyme activity compared to the wild-type protein.

However, the S31F variant displays a lower *V*_max_/*K*_m_ ratio (8.0 nmol DHT h^−1^ mg^−1^ (*μ*mol/L)^−1^ T^−1^) than the wild-type form (14.0 nmol DHT h^−1^ mg^−1^(*μ*mol/L)^−1^ T^−1^), which points toward an enzyme catalytically less efficient. Because the patient was originally referred for hypospadias, it was considered important to exclude genetic alterations in other candidate genes, including the androgen receptor, steroidogenic factor-1, and 17*β*-hydroxysteroid dehydrogenase 3. Molecular screening studies excluded mutations in the *NR3C4, NR5A1*, and *HSD17B3* genes.

## Discussion

The phenotypic spectrum associated with 46,XY DSD due to autosomal recessive mutations in the *SRD5A2* gene is broad, varying from normal female external genital appearance to micropenis, isolated hypospadias, or micropenis associated with hypospadias of varying severity.

In this study, we characterize a molecular defect in a case of 46,XY DSD in which the causative mutation was found to be a novel heterozygous p.Ser31Phe substitution. This mutation appears to increase rather than decrease enzyme activity. To date, approximately 96 different genetic variants of *SRD5A2* have been reported, including 70 missense/nonsense mutations, two gross deletions, 13 small deletions, and six splicing mutations (http://www.hgmd.cf.ac.uk/ac/gene.php?gene=SRD5A2). Most of these variants are pathogenic mutations that affect binding to the cofactor Nicotinamide Adenine Dinucleotide Phosphate or the substrate, resulting in 46,XY DSD due to a deficiency of 5*α*-reductase. With the exception of two single-nucleotide polymorphisms (A49T and V89L), the remaining 60 single-base missense mutations have deleterious effects on enzyme activity, mainly through a loss-of-function effect (Vilchis et al. 2010; Fernandez-Cancio et al. 2011; Maimoun et al. 2011). In this regard, three types of missense mutations have been recognized: (1) those that result in increased enzyme activity, (2) those that display roughly wild-type activity, and (3) those that result in scarce or undetectable enzyme activity (Wigley et al. 1994; Makridakis et al. 2004). The p.Ser31Phe mutation is localized toward the NH2- terminus, within a region considered to serve as the substrate-binding domain of the enzyme. Unlike other proximal single-base substitutions (i.e., L20P, P30L, G32S, G34W, or G34R), which result in nonfunctional enzymes, the p.Ser31Phe mutant retains its reductive capacity as evidenced by the displayed apparent *V*max. An in silico analysis using various bioinformatics methods that predict whether a given nonsynonymous variation may be disease-related (Chan 2013) yielded contradictory results, whereas PolyPhen-2 (http://genetics.bw.harvard.edu/pph2) predicted that p.Ser31Phe might be damaging (score 0.874), PON-P (http://bioinf.uta.fi/PON-P/) and SIFT (http://sift.jcvi.org/) predicted a low pathogenicity for this same mutation. However, the results from expression assays in transiently transfected HEK-cultured cells showed abnormal kinetic characteristics for the p.Ser31Phe mutant, which are consistent with an atypical enzyme activity (Fig. 2). The functional significance of this observation is that, to our knowledge, p.Ser31Phe represents one of the first characterized noninactivating mutations associated with 46,XY DSD. The occurrence of disease-associated monoallelic mutations may not be rare; for example, this appears to be a common mechanism in Kallmann syndrome caused by mutations in *PROKR2* or *PROK2* (i.e., an autosomal recessive mode of disease transmission). Most patients carrying mutations in these genes are identified as heterozygous for missense mutations that impair PROKR2 signaling (Dode and Rondard 2013). Furthermore, monoallelic mutations in the *NR5A1* (SF1) gene are considered a frequent cause of 46,XY DSD. It has been estimated that 5–15% of newborn male patients with genital ambiguity, underandrogenization, partial gonadal disgenesis, and absence of Müllerian derivatives are heterozygotes for mutations in *NR5A1*/SF1 (Köhler et al. 2009). In functional studies, it has been shown that practically all the *NR5A1* mutant fail to transactivate the promoter of SF1-responsive enzymes, including that of CYP11A1, CYP17A1, and HSD3B2 (Camats et al. 2012). Regarding *SRD5A2*, we previously described a gene variant (p.G183S) that increases enzyme activity; however, this mutation was detected in a compound-heterozygous patient harboring a second nonsense mutation (p.P212X) in the other allele (Vilchis et al. 2008). Heterozygous single-base mutations that produce enzymes with abnormal biochemical characteristics (i.e., A52T, P212R, G203R, and H231R) have been identified in a few undermasculinized 46,XY subjects (Russell et al. 1994; Vilchis et al. 2010) and in some boys with isolated hypospadias (Silver and Russell 1999). Two patients with deficiency of 5*α*-reductase and a single-mutant allele are documented (i.e., p.R145W, p.A52T); however, the biochemical consequences of these gene variants were not assessed (Nicoletti et al. 2005; Shabir et al. 2012). Although at least three other activating missense substitutions have been described in *SRD5A2* (p.Val3Ile, p.Phe118Leu, and p.Ala248Val), these were identified from microdissected prostate adenocarcinoma samples and were found to be somatic mutations (Makridakis et al. 2004). To date, none of these three variants have been detected as germline mutations in cases of genital ambiguity or deficiency of 5*α*-reductase (http://www.hgmd.cf.ac.uk/ac/gene.php?gene=SRD5A2). In summary, this report supports the concept that heterozygous missense mutations in *SRD5A2* may induce masculinization defects throughout a noninactivating effect. Likely, the p.Ser31Phe mutation elicited a deregulation of the enzyme activity that led to inadequate levels of DHT to sustain the proper development of external genitalia. Such mechanisms may account for other cases of 46,XY DSD with only one affected *SRD5A2* allele.
